# Educational therapy of adolescent idiopathic scoliosis treated by brace

**DOI:** 10.1186/1748-7161-9-S1-P1

**Published:** 2014-12-04

**Authors:** Jean-Claude Bernard, Julie Deceuninck, Muriele Schneider, Laurence Moisson, Audrey Combey, Rachel Bard, Anne-Lise Nogues, Laurence Burel, Gregory Notin, Lydie Journoud

**Affiliations:** 1 CMCR des Massues, Lyon, France; 2 Ets Lecante, Lyon, France

## Introduction

Adolescent idiopathic scoliosis treated by brace assent with The Patient Educational Therapy (PET) according to the “ARS” in 2013.

## Inclusion

Adolescents between 12 and 15 years old with a tolerance for younger children who are still in adolescent problematic. Evolutional scoliosis diagnosis and orthopedic treatment indication.

## Objectives

Improve compliance.

Improve understanding about scoliosis and its progress.

Preserve quality of life.

Course.

Educational diagnosis during the week of brace adaptation.

PET program is shape by 5 workshop on 1 day: expression group between adolescents, around the brace, experience about daily life with the brace, physical activity with brace, expression group between parents.

## Discussion

The PET objectives engage the adolescent, his parents, his physiotherapist and his physician on the conservative treatment of scoliosis and allow each of them to acquire adaptability capacities and self-care capacities. Evaluation questionnaires completed by the adolescent and his parents are in analysis.

